# A Narrative Review of the Significance of Popular Diets in Diabetes Mellitus Management

**DOI:** 10.7759/cureus.61045

**Published:** 2024-05-25

**Authors:** Sümeyra Şahin Bayram

**Affiliations:** 1 Nutrition and Dietetics, Selçuk University, Konya, TUR

**Keywords:** high-protein diets, low-fat diets, paleolithic diet, gluten-free diet, intermittent fasting, ketogenic diets, plant-based diets, popular diets, diabetes mellitus

## Abstract

Diabetes mellitus is a collection of metabolic disorders marked by elevated levels of glucose in the blood due to irregularities in the generation or functioning of insulin. Medical nutrition therapy and weight loss are crucial elements in the management of diabetes and the prevention of complications. Several diets have become popular over time for the goal of achieving weight loss, but their popularity has declined due to a lack of reliable scientific evidence.

This study classifies popular diets into three categories: diets that manage the composition of macronutrients, diets that restrict specific foods or food groups, and diets that manipulate meal timing. The review includes research studies that investigated the effects of popular diets on the prevention, management, and complications of diabetes.

It is clear that different popular diets can have positive effects on both preventing and treating diabetes and preventing and treating complications related to diabetes. However, it is not practical to determine which diet is the most effective option for preventing or controlling diabetes. Thus, the main focus should be on common underlying factors that support well-being, such as decreasing the intake of refined grains and added sugar, choosing non-starchy vegetables, and giving priority to whole foods over processed foods whenever possible, until there is stronger evidence supporting the specific benefits of different dietary patterns.

## Introduction and background

Diabetes is a long-term and diverse metabolic ailment defined by high levels of sugar in the blood, known as hyperglycemia. This condition arises from either a lack of insulin or the body's reduced response to insulin, resulting in impaired functioning of several organs and impacting multiple systems in the body [1]. The global diabetes population was anticipated to be 537 million in 2021, with projections indicating an increase to 643 million by 2030 and 783 million by 2045 [2]. The American Diabetes Association (ADA) suggests that overweight or obese persons with type 2 diabetes should reduce their energy consumption by following a hypocaloric diet. This dietary approach aims to promote weight loss, which in turn leads to improvements in blood glucose levels [3].

Obesity is a significant and separate risk factor for the development of type 2 diabetes and other diseases associated with weight. These consequences lead to high rates of diseases and death [4]. A 5-15% reduction in weight leads to a notable enhancement in glycemic parameters among patients with both obesity and diabetes mellitus (DM) [5]. The Academy of Nutrition and Dietetics advises making sustainable modifications to lifestyle behaviors, such as decreasing energy consumption, enhancing energy expenditure, and enhancing nutrient density [6]. The ADA advises reducing HbA1c levels to less than 7%, maintaining blood pressure below 130/80 mmHg, and keeping low-density lipoprotein cholesterol (LDL-C) below 100 mg/dL (or below 70 mg/dL for individuals with diagnosed cardiovascular disease (CVD)) in order to minimize the likelihood of microvascular and cardiovascular complications [3].

The primary principles of medical nutrition therapy (MNT) for type 2 diabetes involve the reduction of energy consumption and the careful monitoring of carbohydrate intake [7]. Various popular diets have been promoted for weight loss but often lack reliable scientific data. Common dietary patterns frequently revolve around the incorporation or limitation of various food items or categories [8]. For individuals with DM, modifying their eating behaviors is a crucial and challenging aspect of their lifestyle adjustment. For a long time, specialists have advised people with diabetes to follow a traditional "portion-controlled carbohydrate-containing diet." However, researchers are actively investigating the impacts of various diets on this group [9]. The objective of this review is to assess and compare the efficacy of widely used diets in promoting weight loss and/or managing blood sugar levels in individuals with DM.

## Review

Methods

This study was conducted by searching the databases "PubMed, Web of Science, ScienceDirect, Google Scholar, and Scopus" through Selcuk University Library (using the following keywords alone or in combinations: "diabetes mellitus" "popular diets," "plant-based diets," "vegan diet," "mediterranean diet," ketogenic diet," "very low fat diet," "high protein diet," "paleolithic diet," "gluten-free diet," and "intermittent fasting")

This review categorizes popular diets into three groups: diets that manipulate the content of macronutrients, diets that restrict specific foods or food groupings, and diets that manipulate the time of meals. The analysis studies that investigated the impact of popular diets on diabetes prevention, management, and complications.

Diets based on manipulation of macronutrient content

In order to ensure a long and healthy life, it is necessary to include a combination of these macronutrients in our diet. It remains uncertain whether there exists a specific combination of macronutrients that offers the best possible health benefits. Throughout history, human populations have been able to sustain themselves on diets that have varied significantly in terms of the quantity of macronutrients, when measured as a percentage of energy in the diet [10]. This section analyzed popular diets that differ in macronutrient content and their effects on satiety, reducing hunger, thermogenesis, hormones, metabolic pathways, gene expression, fat storage, and the composition and function of the intestinal microbiome. In this category, there are low- or very-low-fat diets, high-protein diets, and low-carbohydrate ketogenic diets [8,11]. 

Low-/Very-Low-Fat Diets

The traditional method for losing weight, referred to as low-fat (LF) diets, involves consuming less than 30% or 10% of calories from fat or saturated fat. This results in a higher intake of protein or carbohydrates, with the latter form of diet being known as a low-fat high-carbohydrate (LFHC) diet [12]. Models that are classified as very low-fat diets have less than or equal to 10% of their energy intake mostly derived from fat. The Ornish and Pritikin lifestyle programs are well recognized as highly effective very low-fat dietary patterns [13]. 

The Ornish Diet is a dietary pattern that prioritizes consuming unprocessed, plant-based foods with very low levels of fat. This diet highlights mainly vegetables, but also includes fruits, legumes, grains, non-fat dairy products, and egg whites. It is designed to provide 70% of energy from carbs, 20% from protein, and 10% from fat [14]. The Pritikin Diet, initially developed to address cardiovascular disorders by reducing cholesterol and blood pressure, consists of a macronutrient distribution of 75-80% carbs, 10-15% protein, and less than 10% fat. Additionally, it has been advocated as a dietary approach for weight loss and managing blood glucose levels in individuals with prediabetes or type 2 diabetes mellitus (T2DM) [15]. Nevertheless, consuming a greater amount of carbohydrates, which primarily consist of sugar and starch, might trigger the release of insulin and decrease the levels of glucose and triglyceride (TG) in the bloodstream [16]. Insulin promotes the formation of energy reserves, resulting in the accumulation of surplus glucose as adipose tissue which is harmful to weight control [17]. Raised TG levels often coincide with reduced levels of high-density lipoprotein cholesterol (HDL-C) and increased ratios of total cholesterol to HDL-C. Another issue to consider is the development of atherogenic postprandial lipemia that is linked to extremely LF diets, as well as the increase in palmitate levels in circulating TGs caused by elevated fatty acid production [18]. 

High-Protein Diets

High-protein (HP) diets contain a protein level that exceeds 25% of energy intake, equivalent to more than 2.0 grams of protein per kilogram of body weight per day.

Two examples of HP diets include the Atkins and Zone diets [19]. Dr. Barry Sears designed the Zone diet to decrease inflammation caused by nutrition. This diet recommends that 40% of the energy consumed should come from low-glycemic carbohydrates, while proteins and lipids should each contribute 30%. In order to achieve health benefits, it is necessary for each meal to adhere to a protein-to-carbohydrate ratio of 0.75 [20]. 

Clinical trials have found that HP diets can promote weight reduction by increasing diet-induced thermogenesis [21]. Additionally, these diets may be beneficial for improving glycemic control and reducing cardiovascular risk factors in individuals with T2DM [22,23]. However, glucagon is the main hormone that counteracts the effects of insulin. Animal protein intake in persons who are in good health promotes the release of glucagon.

Animal-based protein intake exacerbates the rise in plasma glucagon in patients with diabetes. Consuming a large amount of animal protein can cause insulin resistance by stimulating the release of glucagon in both healthy people and individuals with diabetes, making it harder to control their metabolism [24]. 

Increased protein intake might potentially cause an increase in both the volume and weight of the human kidney, which in turn may lead to the development of glomerular hyperfiltration. An elevated consumption of dietary protein results in increased concentrations of urea and other nitrogenous waste substances, such as blood urea nitrogen (BUN). Elevated levels of BUN enhance the process of protein carbamylation and lead to the production of reactive oxygen species (ROS). Researchers declare that it leads to an increase in oxidative stress, inflammation, endothelial dysfunction, and cardiovascular disorders [25].

Additionally, certain studies have found a correlation between excessive consumption of red meat and an elevation in inflammation and oxidative stress, which causes diabetes. This leads to the activation of inflammatory mediators such as nuclear factor kappa-B (NF-kB) and inflammatory cytokines [26,27]. 

Low Carbohydrate Ketogenic Diets

A low carbohydrate ketogenic diet (LCKD) induces a distinct shift in the body's metabolism. The liver undergoes partial oxidation of fatty acids, resulting in the formation of ketone bodies. Ketosis is the condition in which the body accumulates an excessive amount of ketone bodies that it is unable to digest. A ketogenic diet is characterized by its high fat content and its ability to induce ketosis [28]. Carbohydrate-restricted LCKDs can be categorized into a classical ketogenic diet, Atkins diet, modified Atkins diet, medium-chain triglyceride ketogenic diet, and low glycemic index treatment [29]. 

Several mechanisms have been proposed to explain the connection between a diet high in fat and obesity: (i) A high-fat diet induces a shift in the composition of the intestinal microbiota, resulting in an increase in Firmicutes and a decrease in Bifidobacterium; (ii) Insufficient levels of adenosine monophosphate kinase result in reduced fatty acid oxidation; (iii) The expression of fasting-induced adipose factor triggers the activation of lipoprotein lipase, resulting in the buildup of TG; (iv) Inadequate levels of glucagon-like peptide 1 result in heightened insulin resistance and reduced release of bile acids from the liver; (v) Reduced levels of peptide YY lead to decreased feelings of fullness in those who are obese [16,30,31]. 

The carbohydrate-insulin model of obesity proposes that a high-carbohydrate diet, including large amounts of refined starchy foods and sugar, as commonly consumed in the low-fat diet period, produces postprandial hyperinsulinemia, promotes the deposition of calories in fat cells instead of oxidation in lean tissues, and thereby predisposes to weight gain through increased hunger, a slowing metabolic rate, or both [11]. Insulin is an extremely efficient anabolic hormone that facilitates the absorption of glucose into tissues, limits the release of fatty acids from adipose tissue, hinders the generation of ketones from the liver, and encourages the storage of fat and glycogen. Reducing the consumption of dietary carbs can result in a decrease in the amount of insulin needed, an enhancement in the body's response to insulin, and a drop in the level of glucose in the blood after a meal. From this perspective, LCKD could potentially have a beneficial impact on the treatment of metabolic disorders and the development of obesity [32]. Recent studies have irrefutably demonstrated that implementing an LCKD effectively lowers the HbA1c level in individuals with diabetes. HbA1c is widely recognized as the benchmark for diagnosing and treating diabetes, as well as assessing the extent of oxidative stress [33-35]. During the transition from using carbohydrates to utilizing fat as the primary source of energy (keto-adaptation), individuals may experience symptoms such as weariness, lethargy, and headaches. Adverse consequences of a ketogenic diet encompass dehydration, dyselectrolytemia, hyperuricemia, and hypovitaminosis. In addition, the effectiveness of LCKD may lead to hypoglycemia among individuals with diabetes [36-38]. Hence, it is crucial to diligently observe individuals who are prescribed insulin or antidiabetic drugs. Furthermore, due to the association between diabetes and oxidative stress, the ketogenic diet has a significant drawback in that it reduces the body's ability to counteract oxidative stress by reducing the intake of antioxidants and polyphenols. Studies showing the health benefits of diets based on manipulation of macronutrient content are given in Table 1.

**Table 1:** Studies showing the health benefits of diets based on manipulation of macronutrient content CC: calorie-counting; CD: control diet; CHD: coronary heart disease; CRHP: carbohydrate restricted high protein; FBG: fasting blood glucose; HOMA-IR: homeostatic model assessment insulin resistance; HP: high protein; KD: ketogenic diet; LCKD: low-carbohydrate ketogenic diet; LDL-C: low density lipoprotein cholesterol; LFD: low-fat diet; LGI: low glycemic index; MCD: moderate-carbohydrate diet; MD: mediterranean diet; MGI: moderate glycemic index; MP: moderate protein; PBD: plant-based diet; PC: portion-control; PD: paleolithic diet; PLDC: paleolithic-based low-carbohydrate; RCT: randomized controlled trial; TC: total cholesterol; TG: triglyceride

Author, year (reference)	Type of study	Intervention diet	Duration	Sample size	Participants	Metabolic changes
Otten et al. 2016 [39]	RCT	PD vs. LFD	24 mo	70	Overweight women	Higher decrease in HOMA-IR and liver fat at PD vs. LFD
Hall et al. 2021 [40]	RCT	LCKD vs. PBD	2 wk	20	Overweight	Decrease in FBG, HbA1c, and insulin at LCKD vs. PBD
Raben et al. 2021 [41]	RCT	HP (25 E%) and LGI (<50), HP (25 E%) and LGI (>56), MP (15 E%) and MGI (>56), MP (15 E%) and MGI (>56)	3 y	2326	Overweight/obese	No difference in HbA1c, insulin, and HOMA-IR at intergroup
Delgado-Lista et al. 2022 [42]	RCT	LFD vs. MD	7 y	1002	Adults with CHD	Higher cardiovascular events at LFD vs. MD
Gardner et al. 2022 [43]	RCT	KD vs. MD	12 wk	33	Prediabetes/T2DM	Decrease in TG at KD vs. MD
Shemirani et al. 2022 [44]	RCT	PLCD-CC (25-30 %E carb), PLCD-PC (25-30 %E carb), MCD-CC (40-45 %E carb), MCD-PC (40-45 %E carb)	10 wk	60	Metabolic syndrome	Improvement in FBG, TG, TC, and LDL-C at all groups
Thomsen et al. 2022 [45]	RCT	CRHP (30 E% carb, 30 E% protein) vs. CD (50 E% carb, 17 E% protein)	6 wk	72	T2DM	Decrease in HbA1c and TG at CRPH group

Diets based on restriction of certain foods or food groups

This section presents popular diets that advocate for the complete elimination of certain foods and food groups from one's diet. The rationale behind these diets is that these foods hinder weight loss and have detrimental health effects. Additionally, some of these diets argue that modern nutrition practices may contribute to disease development by promoting dietary patterns followed by people in the distant past. This heading examines the gluten-free (GF) diet, Paleolithic diet, and plant-based diets.

GF Diet

GF diets generally aim to eliminate gluten for celiac disease. The main premise is to exclude wheat, barley, rye, oats, and any goods derived from these grains [46]. The list of choices consists solely of food items that are naturally free of gluten (such as legumes, fruits, vegetables, unprocessed meat, fish, eggs, and dairy products) or alternatives to wheat-based foods that have been specifically produced to be gluten-free or have less than 20 parts per million (ppm) of gluten, in accordance with European regulations [47].

The GF diet has been shown to have several positive effects on the body. It can reduce stress on beta cells, improve their volume, increase insulin sensitivity, and enhance glucose tolerance. Additionally, it can reduce intestinal permeability, promote the growth of Lactobacillus in the colon, and decrease the presence of Akkermansia, Dorea, Clostridium, and Coriobacteriacae. The diet also lowers the levels of pro-inflammatory cytokines and adipokines in the blood, while increasing the presence of anti-inflammatory adiponectin. Furthermore, it regulates lipid metabolism by activating peroxisome proliferator activated-receptor alpha (PPAR-a) and peroxisome proliferator-activated receptor gamma (PPAR-g) in adipose tissue [46,48]. The GF diet also modulates the immune system by reducing the quantities of IFN-gamma produced by CD4+ T helper (Th) cells, interleukin (IL)-22 produced by gamma delta T cell receptors, and TH17 cells. Additionally, it elevates the numbers of immunosuppressant M2 macrophages, reduces insulin secretion, and decreases beta cell stress. There is a debate on whether it could prevent type 1 diabetes mellitus (T1DM) or improve the metabolic profile by preserving the quantity of islets [46,49].

However, fructan-type resistant starches, naturally occurring in wheat, promote a healthy gut microbiome. The impact of the diet on the gut microbiota can potentially provide protection against certain forms of cancer, inflammatory disorders, and cardiovascular diseases [50]. Thus, due to the reduction of fructans in a GF diet, which are naturally found in wheat products and have prebiotic properties, it has the potential to induce adverse alterations in gut health. Additionally, the removal of gluten from GF products alters the macro- and micronutrient composition, as well as the nutritional value. The levels of calcium, magnesium, iron, folate, riboflavin, niacin, vitamin B12, and fiber in diets fall, but their glycemic index, carbohydrate, and fat contents increase [51,52].

Paleolithic Diet

The Paleolithic diet, commonly referred to as a hunter-gatherer diet or stone-age diet, is a presumed dietary pattern that is believed to have been common among people living in the Paleolithic era, which occurred from 2.5 million to 10,000 years ago. The diet promotes the intake of meat, fish, eggs, vegetables, fruits, roots, and nuts, while excluding many produced items like dairy products, oils, cereals, legumes, salt, and processed sugars [53,54].

A typical Paleolithic diet consists of approximately 35% of energy derived from lipids, 35% of energy derived from carbs, and 30% of energy derived from proteins. Thus, the Paleolithic diet generally resembles a diet low in carbohydrates [55]. The Paleolithic diet has the potential to facilitate weight loss, enhance the lipid profile, and reduce blood pressure. Moreover, it implies that the Paleolithic diet may enhance insulin homeostasis and glucose metabolism, rendering it a favorable dietary paradigm for individuals with diabetes [55,56].

However, there is evidence suggesting that consuming dairy products, particularly those high in calcium, can impact the secretion and sensitivity of insulin. This, in turn, impacts the regulation of glucose levels in individuals with prediabetes and T2DM [57,58]. The exclusion of dairy products in the Paleolithic diet could potentially hinder glycemic control. The Paleolithic diet additionally ignores grains and legumes. This scenario also diminishes the consumption of dietary fiber, which plays a crucial role in maintaining proper glucose homeostasis in patients with diabetes.

Plant-Based Diets

Plant-based diets (PBDs) are dietary patterns that prioritize the intake of plant-based foods and exclude most or all animal-based foods. There are various categories of PBDs: pesco-vegetarian diets, which exclude all animal products except for fish and seafood; lacto-ovo-vegetarian diets, which exclude meat and fish but allow dairy products and eggs; and vegan diets, which exclude meat and all animal-derived food products, including honey [59,60].

A diet that is low in fat and based on plants has various effects on nutritional intake and body composition, which might subsequently impact insulin sensitivity. Initially, due to their low fat and high fiber content, these diets usually lead to decreases in the density of energy in the diet and the amount of energy consumed, which are not effectively offset by increased food consumption [61]. The initial large-scale randomized clinical experiment examined the effects of exclusively administering a plant-based (vegan) diet to diabetes patients, comparing it to a conventional diet based on the 2003 ADA guidelines. The vegan group experienced a notable decrease in weight and had considerably lower levels of HbA1c compared to the ADA diet group [62].

Dietary fiber is a crucial part of the vegan diet and provides several benefits to human health. It exerts a beneficial impact on the gut microbiome. The relationship between dietary fiber and gut bacteria entails a process of fermentation. The process of bacterial fermentation of various types of fibers, such as resistant starch, some simple sugars, and polysaccharides, results in the synthesis of short chain fatty acids (SCFAs) such as acetate, propionate, and butyrate. These SCFAs have a crucial role in regulating lipid metabolism, controlling cholesterol and glucose levels, promoting anti-inflammatory responses, supporting immunological activities, and maintaining the integrity of the intestinal barrier [63,64]. Vegan diets are also important in mitigating the harmful impacts of free radicals and ROS by utilizing strong antioxidant abilities. Plants possess vital minerals that are needed for a balanced human diet, as well as various primary and secondary compounds that impact both nutrition and health. Ingesting plant-based foods that are abundant in antioxidants has been linked to a decreased likelihood of developing a range of illnesses, including cancer, cardiovascular disease, and type 2 diabetes [65,66].

A randomized clinical trial was conducted over a period of 16 weeks, involving 75 overweight adults who were randomly assigned to two groups. One group followed a vegan diet, while the other group followed a control diet. The purpose of the investigation was to demonstrate the effects of these diets. The vegan group exhibited a significant enhancement in beta cell activity and fasting insulin sensitivity in comparison to the control group [67]. Another investigation showed that adhering to a low-fat vegan diet led to enhanced regulation of blood sugar levels and reduced reliance on medication among individuals aged 50 years or older with type 2 diabetes. The study suggests that the weight loss impact of the vegan diet may contribute significantly to its benefits on hemoglobin A1C levels, which is a measure of long-term blood glucose control [9].

The other plant-based diets, such as the Mediterranean Diet, dietary approaches to stop hypertension (DASH), the New Nordic Diet, and the Planetary Health Diet, permit the use of limited amounts of meat and animal food products. Mediterranean diets are characterized by a high consumption of plant foods, grains (particularly wheat), legumes, vegetables, and fruits. They have a low intake of animal foods and the consumption of seafood varies depending on the geographical location [68]. Additionally, daily consumed beverages in this diet, such as wine in the Christian north side of the sea and tea in the Muslim south side, contain polyphenols. Researchers propose that plant polyphenols, namely those included in common Mediterranean foods such as olive products, may have the ability to inhibit insulin resistance and the development of type 2 diabetes [69,70,71]. The PREDIMED study, a comprehensive and controlled randomized trial conducted at multiple centers, utilized clinical trial data to demonstrate that a Mediterranean diet supplemented with either extra virgin olive oil or nuts effectively prevented diabetes. This diet was found to be significantly more effective than a low-fat diet, reducing the risk of diabetes by 52% in older individuals with a high risk of cardiovascular disease. The primary factor responsible for this positive outcome was mostly ascribed to the general makeup of the eating pattern, rather than caloric restriction, heightened physical activity, or weight reduction [72]. The ATTICA study reported that adherence to the Mediterranean diet was associated with enhanced fasting glucose homeostasis, insulin levels, and a more favorable insulin resistance index (HOMA) in both those with normal blood sugar levels and those with diabetes. Individuals with a high level of adherence to the Mediterranean diet showed a 15% reduction in baseline glucose and insulin levels, as well as a 27% increase in the HOMA index [73].

The DASH diet is a nutritional plan designed to regulate sodium intake within the range of 1500 milligrams (mg) to 2300 mg per day. It emphasizes the consumption of vegetables, fruits, and low-fat dairy products, along with moderate portions of whole grains, fish, poultry, and nuts [74]. Although the DASH diet was originally created to treat hypertension, its specific combination of nutrients and food groups, as well as its significant health advantages such as enhancing the microbiota, decreasing ROS, and reducing pro-inflammatory cytokine levels, suggest that it may be a beneficial dietary approach for managing diabetes and its complications [75].

The New Nordic (ND) diet was created in 2004, drawing inspiration from the Baltic Sea Diet Pyramid. Its purpose is to promote the consumption of fresh, seasonal, and locally sourced foods among the people of the Nordic countries. The ND diet is centered around consuming fresh, locally sourced fruits and vegetables, fish, and healthy grains. The primary nutritional constituents of ND consist of berries and other fruits, fatty and lean fish, legumes, vegetables (particularly cabbage and root vegetables), and whole grains (including barley, oats, and rye) [76,77]. The SYSDIET intervention involved the division of 200 Nordic patients diagnosed with metabolic syndrome into two groups: an isocaloric NN diet group and a control group following a regular diet. This intervention lasted over a period of 18-24 weeks. The study demonstrated that the isocaloric Nordic diet had beneficial effects on low-grade inflammation and also led to enhancements in patients' lipid profiles [78]. A preliminary investigation was carried out on a group of 30 individuals diagnosed with T2DM. Over a period of 12 weeks, these individuals followed the Nordic diet, which is characterized by a moderate to high intake of carbohydrates. The results of this study indicated a 7% decrease in body weight, improved regulation of fasting plasma glucose levels, as well as enhancements in HbA1c levels and blood lipid profiles [79]. Research studies indicate that the metabolic effects of T2DM are equally influenced by the New Nordic diet, Mediterranean diet, and DASH diet [80].

The release of the EAT-Lancet commission's report in 2019, titled 'Food in the Arthropocene: the EAT-Lancet Commission on healthy diets from sustainable systems', aims to offer a worldwide resolution for both nutritional and environmental sustainability. The committee proposed a global overhaul of food systems, aiming to establish clear guidelines and targets for the sorts of food that should be consumed by humans and the agricultural practices necessary to support human health and environmental sustainability [81,82]. The Planetary Health Diet (PHD), resembling the Mediterranean diet, is predominantly a plant-centric diet with restricted quantities of sugar, saturated fat, and animal-derived items, while being abundant in vegetables, whole grains, nuts, fruits, and legumes. Moreover, specialists recommend that half of the planetary health plate should be comprised of fruits and vegetables, while the other half should mostly consist of plant protein sources, whole grains, unsaturated plant oils, and a restricted quantity of animal protein sources [82]. Using data from the Malmö Diet and Cancer cohort, a study compared adherence to the PHD with the EAT-Lancet index. The study found that individuals with high adherence to the PHD had a 25% lower risk of all-cause mortality compared to those with low adherence [83]. In addition, research studies investigating the relationship between the PHD and the occurrence of type 2 diabetes discovered that individuals who closely adhered to the EAT-Lancet reference diet had a lower risk of developing T2DM [84,85]. Studies showing the health benefits of diets based on restriction of certain foods or food groups are given in Table 2.

**Table 2:** Studies showing the health benefits of diets based on restriction of certain foods or food groups ABD: animal-based diet; BMI: body mass index; CD: control diet; CHD: coronary heart disease; HOMA-IR: homeostatic model assessment insulin resistance; hs-CRP: high sensitivity C reactive protein; KD: ketogenic diet; LCKD: low carbohydrate ketogenic diet; LDL-C: low-density-lipoprotein cholesterol; LFD: low-fat diet; MD: mediterranean diet; OD: omnivore diet; PBD: plant-based diet; RCT: randomized controlled trial; SBP: systolic blood pressure; TC: total cholesterol; TMAO: trimethylamine N-oxide; VD: vegan diet

Author, year (reference)	Type of study	Intervention Diet	Duration	Sample size	Participants	Metabolic changes
Wright et al. 2017 [86]	RCT	PBD vs. CD	24 wk	65	Overweight/obese	Decreasing in TC at PBD vs. CD
Kahleova et al. 2018 [87]	RCT	PBD vs. CD	16 wk	75	Overweight	Increasing in B-cell function and insulin sensitivity at PBD vs. CD
Crimarco et al. 2020 [88]	RCT	PBD vs. ABD	16 wk	36	Overweight	Decreasing in TMAO and LDL-C in PBD vs. ABD
Päivärinta et al. 2020 [89]	RCT	ANIMAL: %70 animal / %30 plant CONTROL: %50 animal / %50 plant PLANT: %70 plant / %30 animal	12 wk	136	BMI: 18.5-35.0 kg/m2	Decreasing in TC and LDL-C in PLANT vs. ANIMAL
Hall et al. 2021 [40]	RCT	PBD vs. LCKD	2 wk	20	Overweight	Decreasing in hsCRP, LDL-C and TC at PBD vs. LCKD
Delgado-Lista et al. 2022 [42]	RCT	MD vs. LFD	7 y	1002	Adults with CHD	Lower cardiovascular events at MD vs. LFD
Gardner et al. 2022 [43]	RCT	MD vs. KD	12 wk	33	Prediabetes/T2DM	Increasing in LDL-C at KD vs. MD
Jenkins et al. 2022 [90]	RCT	Vegan Diet vs. Vegetarian Diet	12 wk	164	T2DM	Similarly but marked decreasing in weight loss, HbA1c, and SBP at both groups
Landry et al. 2023 [91]	RCT	VD vs. OD	8 wk	44	Normal/overweight	Decreasing in LDL-C and insulin at VD vs. OD

Diets based on manipulation of meal timing

This section analyzed intermittent fasting (IF) diets. Disrupting the timing of food intake in relation to light or dark cycles can cause an increase in food consumption. This happens because it disrupts the production of satiety hormones like leptin and ghrelin. Additionally, the timing of food intake can affect how much energy is expended through diet-induced thermogenesis. This can ultimately increase the likelihood of becoming overweight or obese. Scientists have suggested different factors that contribute to the disturbance of the circadian rhythm, which eventually results in worse metabolic health. Furthermore, the interaction between the cycling of feeding/fasting and sleeping/waking behaviors in response to light/dark cycles is recognized as a developing component that influences gut microbiota and contributes to higher levels of obesity [17,92,93,94].

Intermittent Fasting

IF, which encompasses numerous approaches, has recently garnered attention for its impact on body weight and other disorders. Intermittent fasting, like energy restriction diets, limits food consumption by emphasizing the specific time of meals during the day or week [95]. Intermittent fasting diets commonly include alternate day fasting (ADF), which requires fasting for 24 hours every other day, and the 5:2 technique, which entails fasting for 24 hours twice a week and following a very low-calorie diet for two other days of the week. In this strategy, fasting can occur on either consecutive or nonconsecutive days. Time-restricted eating (TRE) is a popular method where fasting happens every day for a variable number of hours. Typically, food occurs within a six-hour period, starting with breakfast in the morning and ending with dinner before 3 p.m. This allows for a fasting period of 14-18 hours [96,97].

ADF generally consists of alternating between a day of feasting and a day of fasting. During the feast day, individuals are allowed to eat ad libitum, meaning there are no limitations on the sorts or amounts of food they can ingest. During fast days, individuals have the option to consume solely water, known as zero-calorie. Alternatively, individuals have the option to ingest approximately 25% of their energy requirements (500-800 kcal) on the fast day, which is referred to as modified ADF. During modified ADF, the meal consumed on the fasting day can be consumed either in one sitting or spread out over the course of the day, without affecting the success of weight loss [98,99].

The 5:2 diet is a variant of ADF where individuals have five days of regular eating and two days of fasting per week. Similar to the ADF approach, individuals are allowed to eat freely and without restriction on the designated feast days. During fast days in the 5:2 diet, individuals typically take around 25% of their energy demands, which amounts to 500-800 kcal. These fast days can be scheduled consecutively or nonconsecutively throughout the week [99,100].

TRE, in contrast, differs from ADF and the 5:2 diet in that it requires individuals to fast for a short period of time every day. More specifically, TRE involves confining the eating window to a specified number of hours per day (usually 4 to 10 h), and fasting with zero-calorie beverages (e.g., water, black coffee, black tea, calorie-free beverages) for the remaining hours of the day. During the eating period, individuals do not need to count calories or monitor food intake in any way [99,101].

Several suggested pathways exist regarding how intermittent fasting may contribute to improved health outcomes [95,100]. The oxidative stress theory posits that reduced energy intake leads to a decrease in the production of free radicals by mitochondria [102]. The second thought is the circadian rhythm theory, which posits that physiological processes occur at the optimal period as determined by evolution. Proper fasting might potentially optimize the peripheral clocks of organs, including the liver, adipose tissue, and skeletal tissue [103]. The final stage is a ketogenic state, which is characterized by an increase in β-hydroxybutyrate levels in overweight individuals who are fasting. Following a period of fasting lasting 6-8 hours, the presence of ketones becomes noticeable [104]. This indicates a transition from storing fat to using fat as an energy source, resulting in a decrease in LDL-C and an increase in HDL-C levels. The transition from utilizing glucose as the primary source of energy to utilizing fatty acids and ketones for energy is referred to as intermittent metabolic switching. Moreover, ketosis facilitates weight loss, as the metabolism of ketones demands higher energy expenditure [97]. Due to their positive effects on weight reduction and insulin resistance, IF diets have the potential to effectively regulate glycemic levels in individuals with type 2 diabetes. Scientific literature has studies demonstrating that intermittent fasting diets, such as alternative day fasting, alternative day modified fasting protocols, and the 5:2 intermittent fasting approach, lead to enhanced insulin sensitivity and reduced dyslipidemia [105-108].

The primary concern related to IF is the potential occurrence of hypoglycemia in individuals who are on antidiabetic treatment, including those who are using insulin (both basal and bolus) and sulfonylureas (including short-acting meglitinides). Additionally, hypotension, dizziness, nausea, sleeplessness, syncope, falling, migraine headaches, weakness or weakness that hinders daily tasks, and intense hunger pangs are all included in the list of adverse effects associated with IF diets [109]. Studies showing the health benefits of diets based on manipulation of meal timing are given in Table 3.

**Table 3:** Studies showing the health benefits of diets based on manipulation of meal timing ADF: alternate day fasting; AUC: area under curve; BMI: body mass index; BW: body weight; CD: control diet; CER: continuous energy restriction diet (1200-1500 kcal/d) followed for seven days per week; CON: control; CR: calorie restriction (70% of energy requirements daily, without time prescription); eTRE: eight-hour eating window from 07:00 to 15:00; FBG: fasting blood glucose; HOMA-IR: homeostatic model assessment insulin resistance; IER: intermittent energy restriction diet (500-600 kcal/d) followed for two non-consecutive days per week; IF: intermittent fasting; iTRE: 30% energy requirements between 08:00 and 12:00 hours and followed by a 20-h fasting period on three nonconsecutive days per week, and ad libitum eating on other days; NOGD: non-oxidative glucose disposal; RCT: randomised controlled trial; SC: standard care; TG: trygliceride; TRE: time restricted eating; TRF: time-restricted fasting; WC: waist circumfrence

Author, year (reference)	Type of study	Intervention Diet	Duration	Sample size	Participants	Metabolik changes
Carter et al. 2018 [110]	RCT	IER vs. CER	12 mo	137	T2DM	Similar decrease in BW and HbA1c at the both groups
Gray et al. 2021 [111]	RCT	IER vs. CER	12 mo	121	Overweight women with previous GDM	Similar decrease in BW, FBG, insulin, HOMA-IR, and HbA1c at the both groups
Andriessen et al. 2022 [112]	RCT	TRE vs. CON	3 wk	14	T2DM	Decreasing in NOGD at TRE vs. CON
Chair et al. 2022 [113]	RCT	ADF, 16/8, TRF, CD	3 wk	101	Overweight/obese with prediabetes	Decreasing in BW, BMI, WC, FBG, and TG at the both intervention groups
Obermayer et al. 2023 [114]	RCT	IF vs. CD	12 wk	46	T2DM	Decreasing in BW and HbA1c at IF vs. CD
Steger et al. 2023 [115]	RCT	eTRE vs. CD	14 wk	90	Obese	Decreasing in BW, FBG, HOMA-IR, and heart rate at eTRE vs. CD
Teong et al. 2023 [116]	RCT	iTRE, CR, SC	18 mo	209	Obese	Greater improvement postprandial glucose AUC, insulin AUC, and TG at month 6 in iTRE vs. CR

## Conclusions

To summarize, upon analyzing the different popular diets discussed in this review alongside the results of randomized controlled studies, it becomes evident that they effectively promote weight loss. Moreover, studies have demonstrated the positive effects of these diets on preventing diabetes and avoiding or managing its associated complications. Various mechanisms, such as energy restriction, manipulation of macronutrients, restriction or elimination of specific foods or food groups, and altering feeding schedules, achieve these effects.

It is not feasible to determine which diet is the optimal choice for preventing or managing diabetes. Research indicates that there is no universally recommended proportion of energy from carbohydrates, protein, and fat for individuals with diabetes or those at risk. Consequently, the distribution of macronutrients should be determined based on a personalized evaluation of an individual's current dietary patterns, preferences, and metabolic objectives. Regular adjustments and sustainability of nutritional treatment recommendations are necessary in response to changes in an individual's living situations, preferences, and disease progression. The primary emphasis should be placed on shared fundamental factors that promote wellness, such as reducing the consumption of refined grains and added sugar, opting for non-starchy vegetables, and prioritizing whole foods over processed foods whenever feasible, until there is more robust evidence supporting the varying advantages of different dietary patterns.
